# An Evaluation of the Novel Biological Properties of Diterpenes Isolated from *Plectranthus ornatus* Codd. In Vitro and In Silico

**DOI:** 10.3390/cells11203243

**Published:** 2022-10-15

**Authors:** Przemysław Sitarek, Tomasz Kowalczyk, Ewelina Synowiec, Anna Merecz-Sadowska, Gabrielle Bangay, Salvatore Princiotto, Tomasz Śliwiński, Patricia Rijo

**Affiliations:** 1 Department of Biology and Pharmaceutical Botany, Medical University of Lodz, ul. Muszyńskiego 1, 90-151 Lodz, Poland; 2 Department of Molecular Biotechnology and Genetics, Faculty of Biology and Environmental Protection, University of Lodz, 90-237 Lodz, Poland; 3 Laboratory of Medical Genetics, Faculty of Biology and Environmental Protection, University of Lodz, Pomorska 141/143, 90-236 Lodz, Poland; 4 Department of Computer Science in Economics, University of Lodz, 90-214 Lodz, Poland; 5 CBIOS—Lusófona University’s Research Center for Biosciences and Health Technologies, 1749-024 Lisbon, Portugal; 6 Instituto de Investigação do Medicamento (iMed.ULisboa), Faculdade de Farmácia, Universidade de Lisboa, 1649-003 Lisbon, Portugal

**Keywords:** *Plectranthus ornatus*, isolated diterpenes, anti-inflammatory activity, DNA damage, in silico studies, mitochondrial copy number, mitochondrial membrane potential, ROS levels

## Abstract

*Plectranthus ornatus* Codd, the genus *Plectranthus* of the Lamiaceae family, has been used as traditional medicine in Africa, India and Australia. Pharmacological studies show the use of this plant to treat digestive problems. In turn, leaves were used for their antibiotic properties in some regions of Brazil to treat skin infections. The present study examines the anti-inflammatory, antioxidant and cytotoxic effects of the halimane and labdane diterpenes (11R*,13E)-11-acetoxyhalima-5,13-dien-15-oic acid (HAL) and 1α,6β-diacetoxy-8α,13R*-epoxy-14-labden-11-one (PLEC) and the forskolin-like 1:1 mixture of 1,6-di-*O*-acetylforskolin and 1,6-di-*O*-acetyl-9-deoxyforskolin (MRC) isolated from *P. ornatus* on lung (A549) and leukemia (CCRF-CEM) cancer cell lines, and on normal human retinal pigment epithelial (ARPE-19) cell line in vitro. Additionally, molecular docking and computational approaches were used. ADMET properties were analysed through SwissADME and proTox-II—Prediction. The results indicate that all tested compounds significantly reduced the viability of the cancer cells and demonstrated no cytotoxic effects against the non-neoplastic cell line. The apoptosis indicators showed increased ROS levels for both the tested A549 and CCRF-CEM cancer cell lines after treatment. Furthermore, computational studies found HAL to exhibit moderate antioxidant activity. In addition, selected compounds changed mitochondrial membrane potential (MMP), and increased DNA damage and mitochondrial copy number for the CCRF-CEM cancer cell line; they also demonstrated anti-inflammatory effects on the ARPE-19 normal cell line upon lipopolysaccharide (LPS) treatment, which was associated with the modulation of *IL-6*, *IL-8*, *TNF-α* and *GM-CSF* genes expression. Docking studies gave indication about the lowest binding energy for 1,6-di-*O*-acetylforskolin docked into IL-6, TNF-α and GM-CSF, and 1,6-di-*O*-acetyl-9-deoxyforskolin docked into IL-8. The ADMET studies showed drug-likeness properties for the studied compounds. Thus, halimane and labdane diterpenes isolated from *P. ornatus* appear to offer biological potential; however, further research is necessary to understand their interactions and beneficial properties.

## 1. Introduction

The plant kingdom is a rich source of bioactive compounds, and medicinal plants remain an important resource in the fight against serious infections and illnesses such as cancer. Despite enormous global advances in modern medicine, such diseases still present a serious problem for public health especially in developing countries [1,2,3,4]. Numerous studies show that both extracts and pure compounds isolated from plants show great biological potential in vitro and in vivo, exhibiting many desirable pharmacological properties [5,6,7,8,9,10].

The *Plectranthus* genus lies within the Lamiaceae family. It comprises about 300 species of evergreen perennials and shrubs, widely distributed in the tropics of Africa from Ethiopia to Tanzania, Asia, and Australia. These species are sources of aromatic essential oils, monoterpenes and sesquiterpenes; in addition, many are edible or grown as ornamental plants. Several species are also used as medicinal herbs, demonstrating anthelmintic, antiseptic, laxative, anti-cancer, and antibacterial effects, among others. They are used as an adjuvant in the treatment of ear infections, intestinal cramps, scorpion stings, nausea and vomiting [11,12,13,14]. 

However, few pharmacological and phytochemical studies have been conducted on these plants to date. One interesting species from this family is *P. ornatus* Codd., commonly known in Brazil as “Boldinho”: a native African plant brought to the Americas by the Portuguese. The plant has historically been used to treat digestive problems, and *P. ornatus* leaf extract has been employed in some regions of Brazil to treat skin infections. Additionally, some farmers in Brazil reported its use in the treatment of bovine mastitis, characterized by physical, chemical, and bacteriological changes in the milk caused by *Staphylococcus aureus.* Phytochemical analysis revealed the presence of secondary metabolites such as labdane, halimane and clerodane diterpenoids [11,12,13,15,16,17]. Additionally, several diterpenes isolated from the plant, such as plectrornatin A, and two labdane derivatives, plectrornatin B and C, demonstrated moderate antimicrobial activity against five *Candida* species and selected bacteria strains [18]. Three further labdane diterpenes were also isolated: 6-*O*-acetylforskolin, 1,6-di-*O*-acetylforskolin, 1,6-di-*O*-acetyl-9-deoxyforskolin or rhinocerotinoic acid [19,20]. Some of these pure compounds were reported to possess interesting anticancer properties: the halimane (11R*,13E)-11-acetoxyhalima-5,13-dien-15-oic acid (HAL) and the labdane 1α,6β-diacetoxy-8α,13R*-epoxy-14-labden-11-one (Plectrornatin C, PLEC) diterpenes induced mitochondrial apoptosis in breast (MCF7) and human pharynx squamous (FaDu) cancer cell lines; unfortunately, such effect was not observed for the forskolin-like 1:1 mixture of 1,6-di-*O*-acetylforskolin and 1,6-di-*O*-acetyl-9-deoxyforskolin (MRC) [21]. Due to the limited number of studies about these species, both phytochemically and biologically, further research is recommended to discover new interesting compounds with potential medicinal effects. 

The present paper continues our ongoing studies on the new biological activities of compounds isolated from *P. ornatus*, including (11R*,13E)-11-acetoxyhalima-5,13-dien-15-oic acid (HAL) and the labdane diterpenes 1α,6β-diacetoxy-8α,13R*-epoxy-14-labden-11-one (PLEC) and forskolin-like 1:1 mixture of 1,6-di-*O*-acetylforskolin and 1,6-di-*O*-acetyl-9-deoxyforskolin (MRC); more specifically, it investigates the biological properties of the compounds in vitro. Also, it explores the potential mechanisms underlying the antioxidant and anti-inflammatory properties via binding free energy calculations and molecular dynamics simulations.

## 2. Materials and Methods

### 2.1. Plant Material and Isolation of Compounds from Plectranthus Ornatus

**Figure 1:**
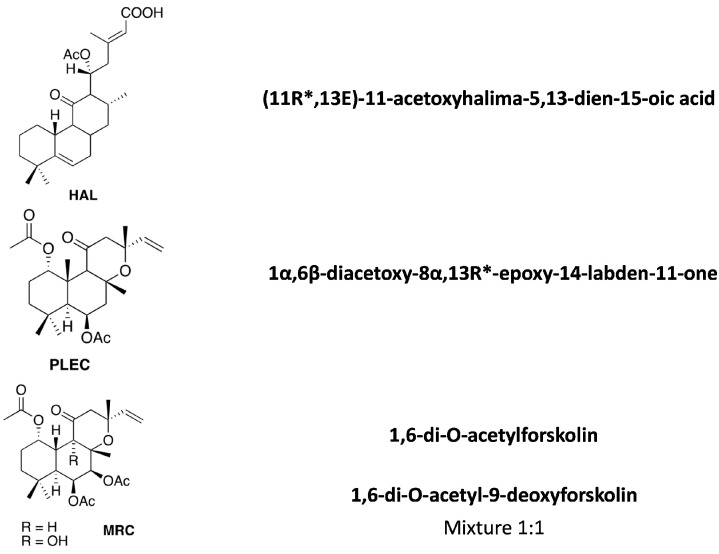
Diterpenes isolated from the described *Plectranthus ornatus* Codd. plant.

*Plectranthus ornatus* Codd. plant derived from South Africa and was cultivated in Instituto Superior de Agronomia campus (Lisbon). Voucher specimens were deposited in the Herbarium of the Botanical Center of the “Instituto de Investigação Científica Tropical”, Lisbon (ref. C. Marques, S/N°LISC). The compounds used for screening had previously been isolated during biased tests [18,19,20]. The structure of the bioactive metabolites was determined based on physicochemical data (melting point, specific rotation), mainly UV, IR, 1D- and 2D- spectroscopic data NMR (^1^H and ^13^C), mass spectrometry, elemental analysis and comparison with bibliographic data. The isolated compounds are shown in Figure 1.

The tested *Plectranthus* plant compounds were isolated and characterized as previously described at the CBIOS Laboratory (Natural Bioactives Lab—Bio.Natural@CBIOS) (Universidade Lusófona, Lisbon, Portugal) [18,19,20].

### 2.2. Cell Cultures

The human lung adenocarcinoma A549 (CCL-185; ATCC, Manassas, VA, USA) cell lines were cultured in DMEM (Dulbecco’s Modified Eagle Medium) medium containing 10% fetal bovine serum (FBS), 100 U/mL penicillin, 100 μg/mL streptomycin in 75 cm^2^ tissue culture flasks under a humidified 5% carbon dioxide (CO_2_) and 95% air atmosphere at 37 °C. Passaging was carried out at 80–90% confluence using 1×TrypLE™ Express Enzyme (Gibco™). The human T lymphoblast CCRF-CEM (CCL-119; ATCC) cell lines were grown in RPMI 1640 medium (supplemented in the same way as described above) at 37 °C in air containing 5% CO_2_ and 100% relative humidity. Studies on anti-inflammatory properties were conducted on human-derived retinal pigment epithelial cells (ARPE-19; CRL-2302; ATCC). The cells were grown in Dulbecco’s Modification of Eagle’s Medium (DMEM)/Ham’s F-12 50/50 mix medium (L-glutamine, antibiotics and FBS were added at the same concentrations as in the above cell lines) on 75 cm^2^ cell culture flasks to approximately 75–85% confluence and after reaching confluence the cells were washed with DPBS (Gibco™), detached from the flasks by a brief treatment with 1×TrypLE™ Express Enzyme. 

### 2.3. Cell Viability

The tetrazolium (MTT) based colorimetric assay was performed to test the cytotoxic activity of the HAL, PLEC and MRC (for A549 and CCRF-CEM cell lines), as per the methodology described by [9]. The cells were treated with the following concentrations of each compound in the range 0–100 µg/mL. The inflammatory process was initiated with 1 μg/mL LPS for the ARPE-19 cell line. After the treatment period (24 h), 10 µL of the MTT solution (5 mg/mL) were added to each well and the plates were incubated at 37 °C for three hours. Then, DMSO (100 µL) was added to solubilize the formazan products and the plate was kept in a shaker for 5 min. The optical density (OD) of each well was measured at 570 nm (reference at 630 nm) using a Bio-Tek Synergy HT Microplate Reader (Bio-Tek Instruments, Winooski, VT, USA). The concentrations of tested compounds required to inhibit cell growth by 50% (IC_50_) were calculated by generating dose-response curves with the software package GraphPad Prism 8.2.1 for Windows (GraphPad Software Inc., La Jolla, CA, USA). The IC_50_ concentration for HAL and PLEC was used for further studies, while the highest tested concentration was used for MRC. Cell survival was calculated using the following equation:

Cell survival (%) = (OD of treated sample)/(OD of untreated sample) × 100

### 2.4. Measurement of Intracellular ROS Level

The cell-permeable fluorogenic probe DCFH-DA (2′7′-dichloro-dihydro-fluorescein diacetate) was used to estimate ROS level [22]. In brief, A549 cells were seeded at a density of 1 × 10^4^ cells/well and CCRF-CEM cells 1 × 10^5^ cells/well in 96-well plate fluorescence. Cells were then incubated with 5 μM DCFH-DA for 45 min in a humidified 5% CO_2_ atmosphere at 37 °C (New Brunswick Galaxy^®^ 170R CO_2_ Incubator, Hamburg, Germany), washed in HBSS, and then treated with the indicated concentrations of HAL, PLEC and MRC for 1, 2, 12, 24 and 48 h. Fluorescence readings were taken at 485/20 nm excitation and 528/20 nm emission in a fluorescence plate reader (Bio-Tek Synergy HT Microplate Reader).

### 2.5. Mitochondrial Membrane Potential (MMP; Δψ)

MMP was assessed with a microplate reader using the Mitochondrial Membrane Potential Probe (JC-1 Dye; Invitrogen™, Carlsbad, CA, USA) [23], according to the protocol described in our previous publications [9,24,25,26]. 

### 2.6. Quantitative Assessment of Mitochondrial DNA Copies

Relative levels of mitochondrial DNA (mtDNA) and nuclear DNA (nDNA) were determined using quantitative Real-Time PCR (qRT-PCR), as previously described [9,24,25,26]. The primer sequences, the composition of the reaction mixture for qRT-PCR and the thermal cycling conditions are described in our previous studies [21]. Briefly, HAL, PLEC and MRC treated cell suspensions (2 × 10^6^ cells) were collected by centrifugation and subsequently total genomic DNA (nuclear and mitochondrial) was isolated using the QIAamp DNA Mini Kit (QIAGEN, Mississauga, ON, Canada). The relative mtDNA copy number was determined through simultaneous assessment of the nuclear genes: *SLCO2B1* and *SERPINA1*, and the mitochondrial genes: *ND1* and *ND5* using CFX96 Touch Real-Time PCR Detection System (Bio-Rad, Hercules, CA, USA). The threshold cycle (Ct) of each sample was determined for each gene and the mtDNA copy number using nDNA as the standard was computed using the following equation: 2^ΔCt1 and Ct2^, where ΔCt1 is the difference in the Ct values for the ND1/SLCO2B1 pair (ΔCt1 = Ct for *SLCO2B1* − Ct for *ND1*); ΔCt2 is the difference in the Ct values for the ND5/SERPINA1 pair (ΔCt2 = Ct for *SERPINA1* − Ct for *ND5*).

### 2.7. Comet Assay

In this study, the comet assay was performed at pH > 13, according to Singh et al. (1988) with later modifications [27]. To examine oxidative DNA damage, two repair endonucleases human 8-oxoguanine DNA glycosylase (hOGG1) and Endonuclease III (Nth) were used (New England Biolabs, Ipswich, MA, USA), according to Smith et al. [28,29]. Briefly, the cell samples (approximately 5 × 10^4^ cells for each slide) were centrifuged (200× *g*, 5 min) and next cell pellets were gently mixed with 50 µL of 0.75% low melting point agarose in PBS cooled to 37 °C and spread onto a microscope slides beforehand precoated with 0.5% normal melting point agarose. The gels were covered with a coverslip and allowed to solidify on a cold plate for 10 min. Thereafter, the coverslips were removed, and the slides were immersed in a chilled lysis solution (pH 10) consisting of 2.5 mM NaOH, 10 mM TRIS, 100 mM EDTA, 1% Triton X-100 and incubated at 4 °C for 1 h. After lysis, the slides (only enzyme-treated samples) were washed three times (5 min each wash) with the enzyme incubation buffer (40 mM HEPES, 0.1 M KCl, 0.5 mM EDTA, 0.2 mg/ml BSA, pH 8.0 with KOH) at room temperature. 50 µL of the enzyme solution—0.2 U hOGG1 (New England Biolabs, Ipswich, MA, USA) and 0.2 U Nth (New England Biolabs) per sample or buffer alone, as control, was then placed onto the gel surface and covered with a coverslip. The enzyme-treated samples and controls were incubated in a humid chamber at 37 °C for 1 h. Following incubation and removal of the cover slip, the slides were immersed in an electrophoresis tank, the DNA was allowed to unwind for 20 minutes in the electrophoresis buffer (300 mM NaOH and 1 mM EDTA, pH > 13). Electrophoresis was performed in the same buffer at 4 °C for 20 minutes at an electric field strength of 0.73 V/cm (300 mA). In the final stage, the slides were rinsed in water, strained and stained with 1 µg/mL DAPI for 1 h in the dark. Fifty cells were randomly selected to determine DNA in the tail of comets (% tail DNA) which was measured through a fluorescence microscope (Nikon, at 200× magnification) and LUCIA Comet Assay™ software v.5.41 (Laboratory Imaging, Praha, Czech Republic). For oxidative DNA damage, the results obtained for hOGG1/Nth were normalized by subtracting the level of DNA damage observed for the buffer alone and HAL, PLEC and MRC treated samples but not treated with the enzyme hOGG1 and Nth, respectively. All the experiments were performed in triplicate.

### 2.8. mRNA Gene Expression

RNA was extracted from normal cell line ARPE-19 incubated first with LPS, and then with HAL, PLEC and MRC for 24 h (in the appropriate concentrations—50 µg/mL). cDNA was synthesised using the High-Capacity cDNA Reverse Transcription Kit (Applied Biosystems™). IL6, IL8, TNFα and GM-CSF mRNA gene expression was examined by RT-qPCR methods using TaqMan^®^ Gene Expression Assays (Life Technologies, Carlsbad, CA, USA), in accordance with the manufacturer’s recommendation. Relative basal expression of each mRNA was calculated by the comparative Ct method (2-ΔCt model) [30] and normalized to the mean 18S rRNA level (reference gene), where ΔCt = Ct target mRNA − Ct of 18S rRNA mRNA. 

### 2.9. ADMET (Absorption, Distribution, Metabolism, Excretion, and Toxicity) Prediction

Physicochemical properties, lipophilicity, water solubility, pharmacokinetics and drug-likeness data were obtained by the SwissADME website (http://www.swissadme.ch/index.php accessed on 12 September 2022). Oral toxicity prediction results, as well as the estimation of the hepatotoxicity, carcinogenicity, mutagenicity and cytotoxicity were predicted by ProTox II website (https://tox-new.charite.de/protox_II/index.php?site=compound_input accessed on 12 September 2022). In addition to the analysis of labdane diterpenes, gemcitabine was used as the positive control.

### 2.10. Computational Studies of Free Radical-Scavenging Properties

Density Functional Theory (DFT) is a tool to provide important information of interest in biological science, including the free radical scavenging activity of phytochemicals. The DFT is known for the optimization of ground state geometries (S0) of molecules. In the current study, S0 geometry optimizations were executed at B3LYP/def2-TZVP level by ORCA. Avogadro can be used to visualize molecular orbitals [31].

### 2.11. Molecular Docking Studies

Blind molecular docking was performed to find possible interactions between the set of four ligands and four target proteins. The plant-derived compounds, *viz.* A (HAL), B (1,6-di-O-acetylforskolin), C (1,6-di-O-acetyl-9-deoxyforskolin) and D (PLEC), were structured using Advanced Chemistry Development/ChemSketch (ACD/ChemSketch) freeware [32]. The files were saved in .mol file format and converted into .pdbqt by Open Babel: The Open Source Chemistry Toolbox [33]. The three-dimensional (3-D) structure of IL-6 (PDB ID: 1ALU), IL-8 (PDB ID: 5D14), TNF-α (PDB ID: 1TNF), GM-CSF (PDB ID: 2GMF) were obtained from the Research Collaboratory for Structural Bioinformatics Protein Data Bank (https://www.rcsb.org/, accessed on 25 August 2022). These proteins were prepared by deleting the water molecules and the unspecified atoms, repairing missing atoms, and adding atoms of polar hydrogens and the charges. The .pdb file format was created and the analysis was conducted using AutoDock 4.2 [34]. Coordination of the center of grid box (X, Y, Z) and spacing (angstrom) were as follow IL-6: X (2.535), Y (−19.89), Z (8.99) and 0.40 Å; IL-8: X (2.386), Y (17.136), Z (−3.85) and 0.30 Å; TNF-α: X (20.063), Y (49.593), Z (40.02) and 0.55 Å; GM-CSF: X (20.602), Y (19.724), Z (−16.575) and 0.60 Å. The analysis included grid size at 126 × 126 × 126 points. The docking studies used the Lamarckian Genetic Algorithm [35]. Genetic algorithm runs were set at 100. The binding energies were computed after the preparation of ligand-protein conformations and ranked according to their binding affinities. The surfaces were graphically analyzed using Discovery Studio Visualizer 4.1 client (https://discover.3ds.com/discovery-studio-visualizer-download, accessed on 25 August 2022).

### 2.12. Statistical Analyses

All statistical analyses of differences between compounds (HAL, PLEC, MRC) were performed in Prism v. 5.00 for Windows (GraphPadSoftware, Inc., San Diego, CA, USA) using ordinary one-way ANOVA followed with Dunnett’s multiple comparison test. The results were expressed as means with standard deviation (SD). The studies were conducted in triplicate, n ≥ 3. 

## 3. Results

### 3.1. Cell Viability after Treatment of Compounds HAL, PLEC and MRC

**Figure 2:**
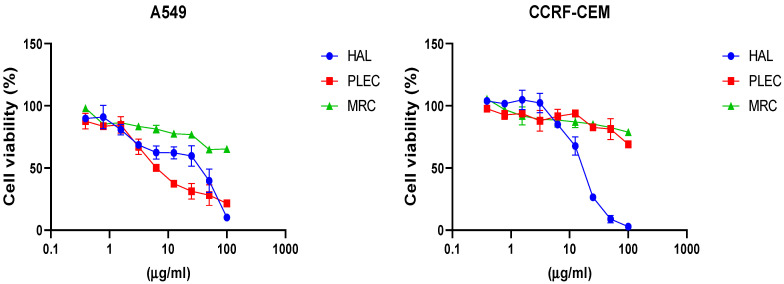
Effects of HAL, PLEC and MRC on A549 and CCRF-CEM cell viability, as determined by the MTT assay after 24 h. Values represent the means ± SD as a percent (%) of control. Values on the *X*-axis are shown as log10.

**Figure 3:**
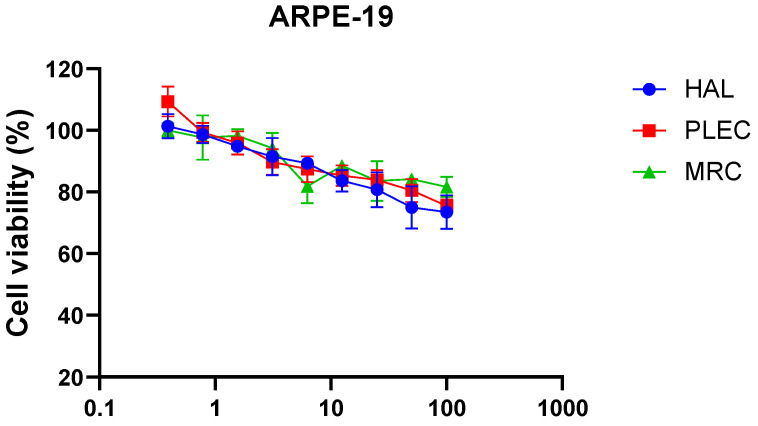
Effects of HAL, PLEC and MRC on the viability of ARPE-19 cells as determined by the MTT assay after 24 h. Values represent the means ± SD as percentage (%) of control values. Values on the *X*-axis are shown as log10.

The cytotoxic effects of HAL, PLEC and MRC were determined in the A549 and CCRF-CEM cell lines. It was found that HAL and PLEC had cytotoxic effects against the A549 cell line with IC_50_ of 19.38 µg/mL and 8.616 µg/mL, respectively. MRC did not show any cytotoxic effect in the tested concentration range (0.39–100 µg/mL). For the CCRF-CEM line, only HAL showed cytotoxic activity with IC_50_ = 16.52 µg/mL. The results are shown in Figure 2. Additionally, no significant decrease in survival was observed for the normal ARPE-19 cell line after treatment with the tested compounds (Figure 3), similarly to what was observed for the tested compounds and LPS (data not shown). 

### 3.2. Measurement of Intracellular ROS Production after Treatment with HAL, PLEC and MRC

**Figure 4:**
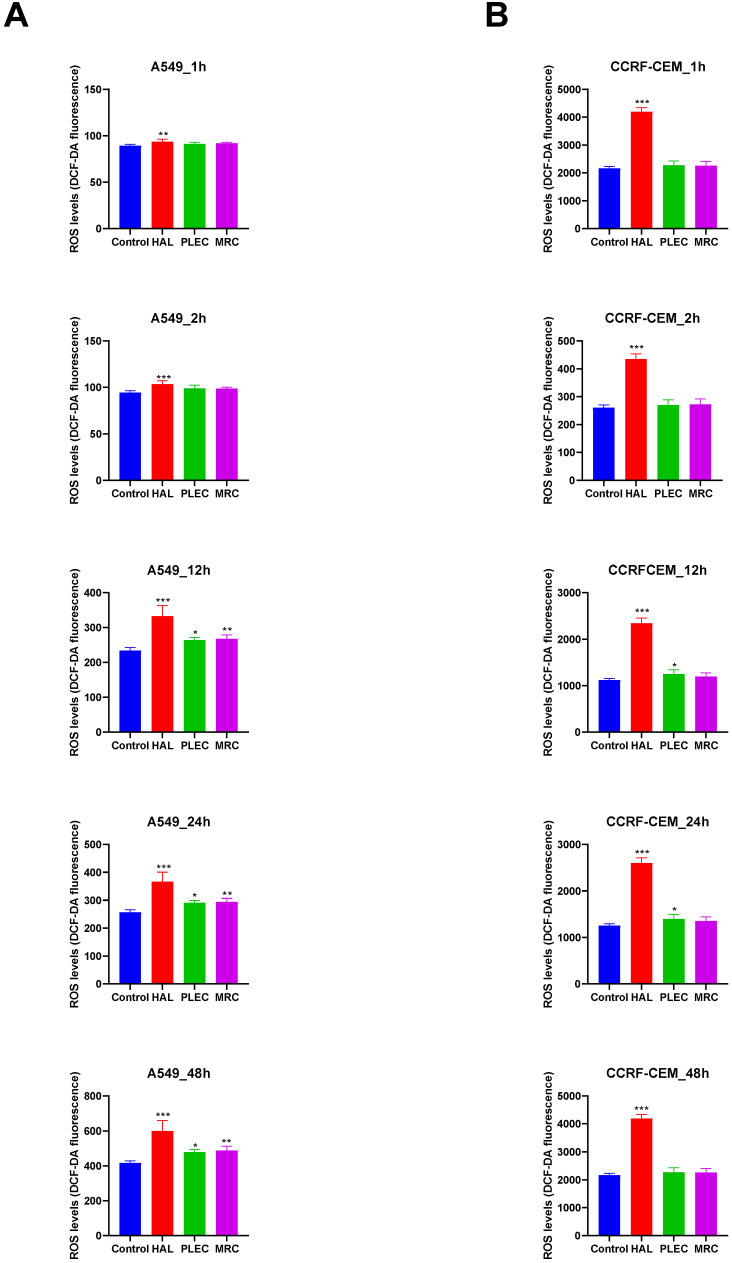
Formation of reactive oxygen species (ROS) in A549 (**A**) and CCRF-CEM (**B**) cells treated with HAL, PLEC and MRC for 1, 2, 12, 24 and 48 h. The results represent means ± SD. * *p* < 0.05, ** *p* < 0.01, *** *p* < 0.001 vs. control cells.

The ROS levels in the CCRF-CEM and A549 cells were measured after 1, 2, 12, 24 and 48-h treatment with HAL, PLEC and MRC. After one-hour incubation of A549 cells with HAL, the levels of ROS were significantly higher (*p* < 0.01) than that of the control cells, and this effect was maintained for up to 48 h (Figure 4A). Furthermore, after 12 h, the cells had higher ROS levels after treatment with PLEC and MRC (Figure 4A); this effect was maintained for up to 48 h. 

After one hour of incubation of the CCRF-CEM cell line with HAL, a significant increase in ROS levels was observed and maintained for up to 48 h (Figure 4B). After 12 h of PLEC treatment, CCRF-CEM cells displayed higher ROS production which was still measurable up to 24 h later (Figure 4B). 

### 3.3. Mitochondrial Membrane Potential

**Figure 5:**
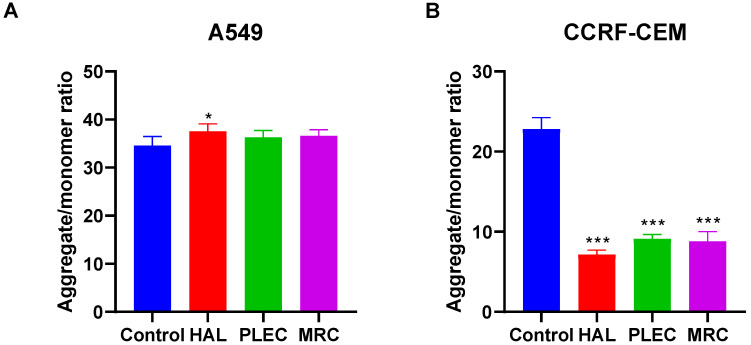
Changes in MMP of A549 (**A**) and CCRF-CEM (**B**) cells incubated with HAL, PLEC and MRC for 24 h. MMP is expressed as ratio of 530 nm/590 nm to 485 nm/538 nm (aggregates to monomer) fluorescence as quantified with a fluorescent plate reader after JC-1 staining. Results are presented as means ± SD. * *p* < 0.05, *** *p* < 0.001 vs. control cells.

The MMP was measured in the A549 and CCRF-CEM cell lines following 24 h incubation with HAL, PLEC and MRC. In the A549 cell line, only HAL increased MMP (Figure 5A). However, in the CCRF-CEM cell line, all tested compounds (HAL, PLEC and MRC) decreased MMP (Figure 5B). 

### 3.4. Mitochondrial Copy Number

**Figure 6:**
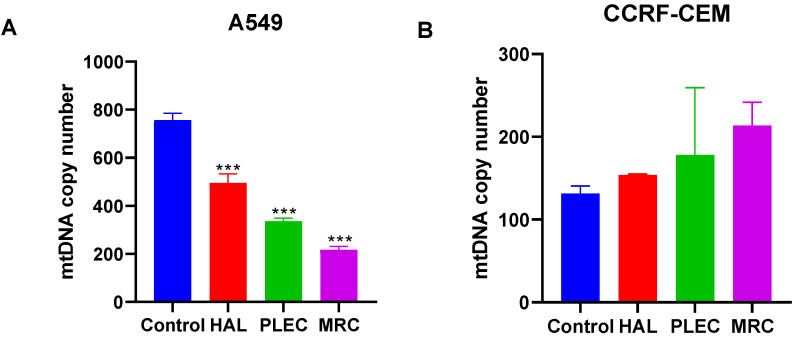
Mitochondrial DNA copy number in A549 (**A**) and CCRF-CEM (**B**) cells measured by real-time quantitative PCR. The cells were exposed to HAL, PLEC and MRC for 24 h and then the DNA was extracted. Values are expressed as means ± SD. *** *p* < 0.001 vs. control cells.

The A549 cells demonstrated a lower mtDNA copy number than control cells after 24 h of incubation with HAL, PLEC and MRC (Figure 6A). In turn in CCRF-CEM cells, there were no statistically significant differences in mtDNA copy number between treated and untreated cells (Figure 6B).

### 3.5. DNA Damage by Comet Assay

**Figure 7:**
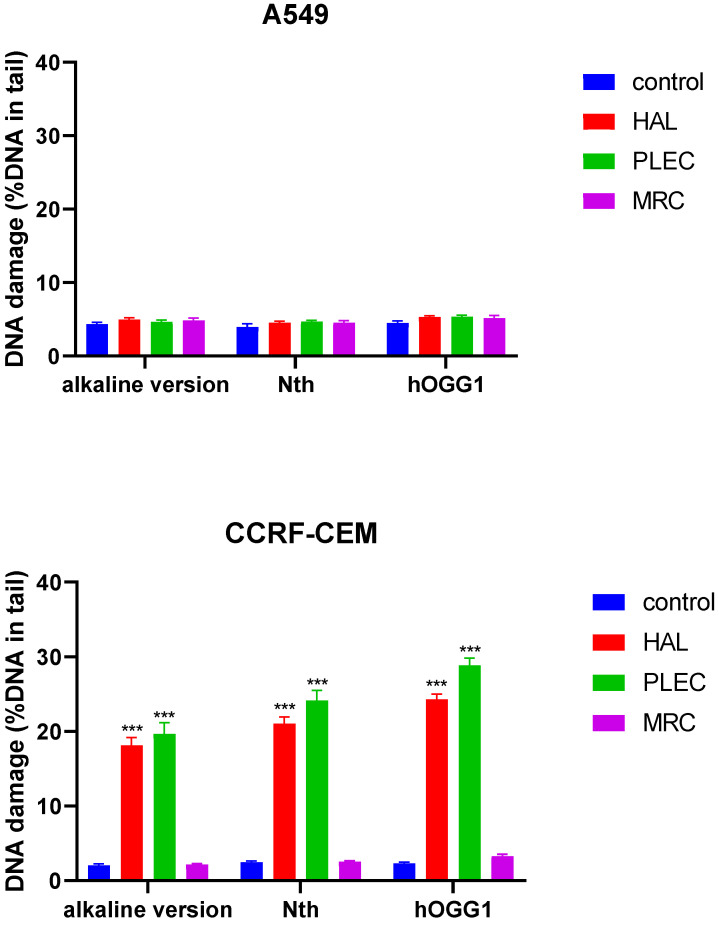
Mean level of DNA strand breaks, alkali-labile sites and oxidative DNA damage induced by HAL, PLEC and MRC in of A549 and CCRF-CEM cells, measured as mean % comet tail DNA in alkaline comet assay. Error bars denote SEM, *** *p* < 0.001 vs. control cells.

The level of DNA damage induced by HAL, PLEC and MRC in A549 and CCRF-CEM cells was determined using an alkaline version of the comet assay to measure the amount of DNA alkali label sites and strand breaks. Significantly higher DNA damage was noticed after treatment of the CCRF-CEM cells with HAL and PLEC (*p* < 0.001) (Figure 7). The extent of the oxidative DNA damage was determined using a modified comet assay with two glycosylases: hOGG1 (excising oxidized purines) and Nth (removing oxidized pyrimidines). In both cases, the damage was significantly higher in CCRF-CEM cells treated with HAL and PLEC than in controls (*p* < 0.001) (Figure 7). However, no statistically significant differences in the level of DNA damage, i.e., DNA breaks or oxidative damage, were found in A549 cells treated with all compounds vs. control cells. Representative images of comets have been added as Supplementary material Figure S1.

### 3.6. Gene Expression

**Figure 8:**
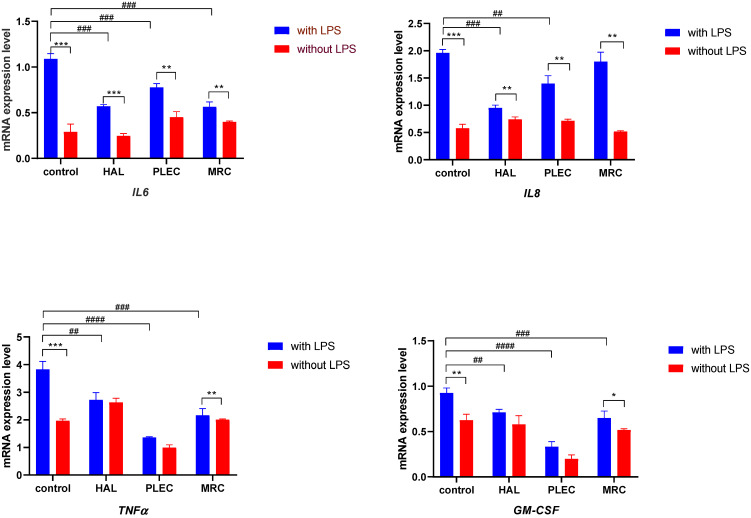
Gene expression (*IL6*, *IL8*, *TNFα* and *GM-CSF*) for ARPE-19 cell lines after one-hour pre-treatment of LPS and 24 h treatment with compounds (HAL, PLEC and MRC). The *18S RNA* was used as a reference gene. The relative expression of mRNA was calculated by the 2-ΔCt method (where ΔCt is the value obtained by subtracting Ct of *18S rRNA* mRNA from Ct of *IL6*, *IL8*, *TNFα* and *GM-CSF* mRNAs, respectively). Data are shown as means ± SD. Statistical differences between groups (treated LPS vs. untreated) at * *p* < 0.05, ** *p* < 0.01 and *** *p* < 0.001 are indicated by stars above the bars. ^##^ *p* < 0.01, ^###^ *p* < 0.001 and ^####^ *p* < 0.0001 cells treated with LPS followed by the appropriate compound relative to control cells (only LPS treated).

mRNA expression of the pro-inflammatory cytokines *IL6*, *IL8*, *TNFα* and *GM-CSF* was determined after 24 h treatment with HAL, PLEC and MRC of ARPE-19 cells, previously undergone one-hour pre-incubation with LPS. All the tested genes in the LPS-stimulated cells showed changes in the expression upon treatment with all the tested compounds, compared to controls (Figure 8).

### 3.7. ADMET Prediction

The estimation of the physicochemical properties (lipophilicity, water solubility, pharmacokinetics, and drug-likeness) of the diterpenes, performed using the SwissADME online server, is presented in Table 1. Gemcitabine was used as the positive control.

Good bioavailability of the compounds can be evidenced by molecular weight ranging between 150 and 500 g/mol, no more than 9 rotatable bonds, the fraction of carbons in the sp^3^ hybridization ranging between 0.25 and 1, TPSA between 20 and 130 Å2, logPo/w values between −0.7 and 5.0 and LogS values between 0 and 6. All the analyzed labdane diterpenes satisfied those rules. However, compounds under investigation displayed only low to moderate solubility, since LogS values ranging between −10 and −6 indicate low solubility and range between −6 and −4 for moderate solubility. The contribution of hydrogen bond donors and acceptors was also considered, as they can significantly influence solubility and permeability through cell membranes. However, the tested diterpenes possess an acceptable number of such groups, ranging within the recommended interval [36,37]. In addition, Lipinski’s rule of 5 was also used to evaluate the bioavailability of the compounds; in particular, the following criteria were taken into consideration: molecular mass less than 500 g/mol, no more than 5 hydrogen bond donors and 10 hydrogen bond acceptors, the octanol-water partition coefficient (log P) not higher than 5. Based on this rule, all the labdane diterpenes displayed favorable physicochemical drug-like properties. The contribution of the number of hydrogen bond donors and acceptors was also significant for the solubility, since too many of them can in turn lead to impaired permeability through cell membranes. The tested diterpenes possess acceptable amount of such groups [36,37].

Computational evaluation of the pharmacokinetic properties of diterpenes showed high gastrointestinal absorption for all compounds. P-glycoprotein (P-gp) is an important factor in multidrug-resistant phenotypes in cancer. P-gp prevents cellular uptake of a large number of structurally and functionally diverse compounds, including most cancer therapeutics and, in this way, can cause multidrug resistance (MDR) [38]. Two of the tested diterpenes are not P-gp substrates. In addition, inhibition of enzymes belonging to the cytochrome P450 (CYP) system by anticancer drugs might lead to adverse drug reactions, multiple-drug resistance, and drug-drug interactions [39]. Most of the analyzed diterpenes do not inhibit CYP activity. Additionally, the lower LogKp value, the lower permeability through the skin indicated. Therefore, analyzed compounds are estimated to have acceptable skin permeability. Also, all the compounds under investigation resulted not able to permeate the blood-brain barrier (BBB) and, as a consequence, are less likely to give central nervous system side effects. Furthermore, most of the compounds were predicted to have low toxicity (predicted toxicity class V: harmful if swallowed, 2000 < LD50 < 5000), and only PLEC showed an LD50 at 100 mg/kg. The prediction accuracy was around 70% for all the compounds. 1,6-di-O-acetylforskolin was the only one likely to show immunotoxicity, while hepatotoxicity, carcinogenicity (apart from HAL), mutagenicity, and cytotoxicity were predicted to be of no concern (Table 2).

### 3.8. Computational Studies of Free Radical-Scavenging Properties

Table 3 present the highest (HOMO) and lowest (LUMO) occupied molecular orbital energies, HOMO-LUMO energy gaps (Egaps), ionization potential (IP) and electron affinity (EA). Reactivity descriptors were calculated as follows: IP = −EHOMO; EA = −ELUMO.

**Figure 9:**
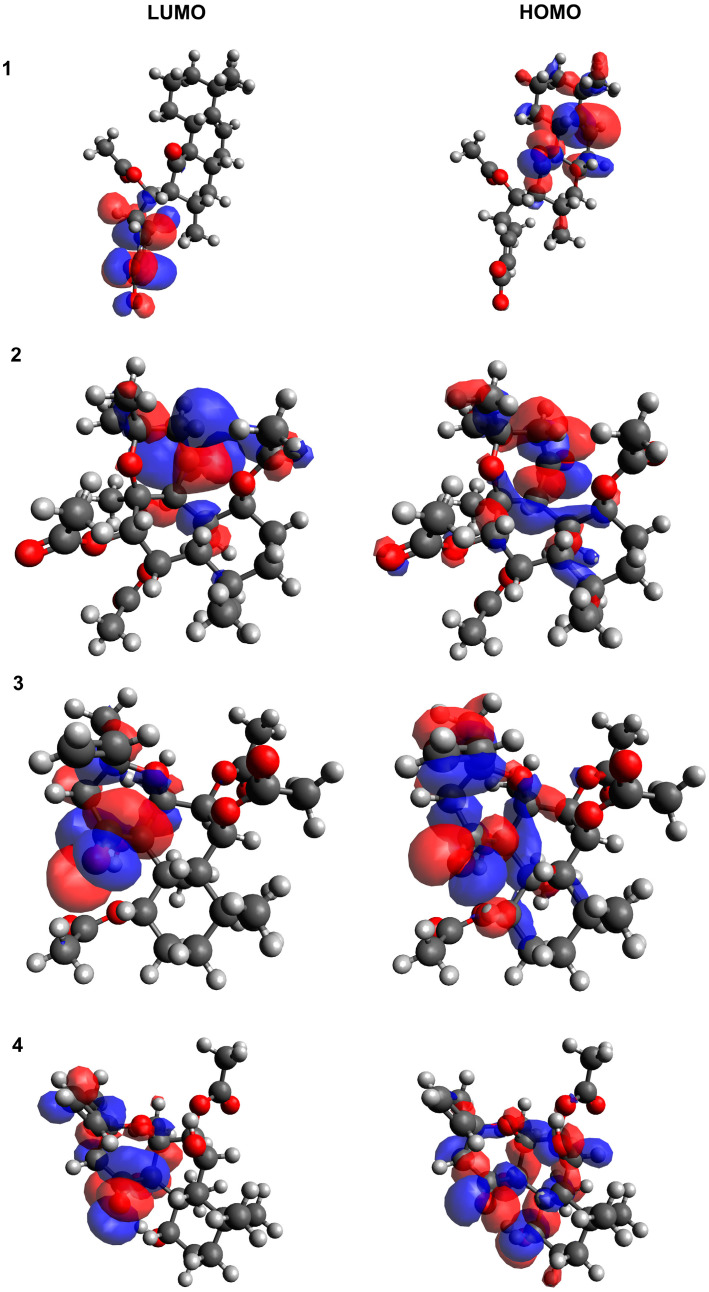
The HOMO/LUMO electron densities in molecules of A–D studied compounds. (**1**) HAL; (**2**) 1,6-di-*O*-acetylforskolin; (**3**) 1,6-di-*O*-acetyl-9-deoxyforskolin; (**4**) PLEC.

The HOMO and LUMO electron densities for diterpenes are shown in Figure 9. The HOMO-LUMO Egaps is an important descriptor of the chemical and biological activity of molecules. A smaller gap value indicates a more chemically active molecule. In this study, caffeic acid and phenol were used as positive and negative standards, respectively. Phenol was found to exhibit a similar HOMO-LUMO Egaps value (5.861 eV) to 1,6-di-*O*-acetylforskolin, 11,6-di-*O*-acetyl-9-deoxyforskolin and PLEC, which may indicate their similar reactivity properties and absence of antioxidant potential. Compound HAL (4.794 eV) exhibited moderate reactivity properties. In addition, caffeic acid, a known antioxidant, has the lowest HOMO-LUMO Egaps value (4.127 eV) and the best reactivity.

### 3.9. Molecular Docking

**Figure 10:**
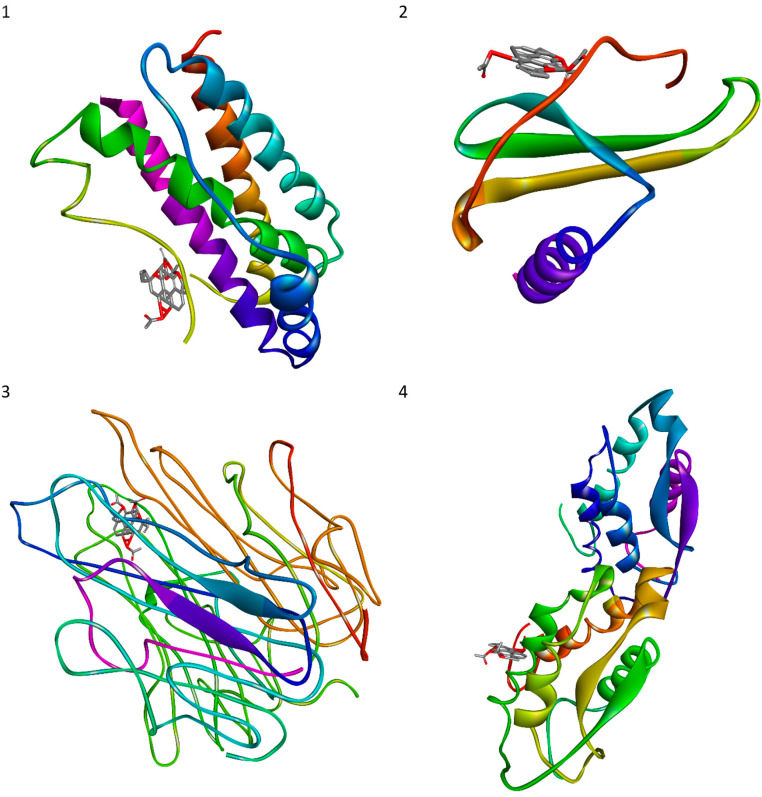
Diterpenes dock into targeted proteins with the lowest energy: (**1**) IL-6 with 1,6-di-*O*-acetyl-9-deoxyforskolin; (**2**) IL-8 with 1,6-di-*O*-acetylforskolin; (**3**) TNF-α with 1,6-di-*O*-acetyl-9-deoxyforskolin; (**4**) GM-CSF with 1,6-di-*O*-acetyl-9-deoxyforskolin.

Molecular docking indicates that the ligand is correctly absorbed and binds the target protein. The lowest docking energy is associated with the most significant interaction between ligand and target protein and the highest binding affinities. Autodock 4.2 was applied to evaluate affinity, binding conformation, best ligand and target protein orientation. For four ligands, only those that had the highest docking score were selected. The strength of binding, the number of hydrogen-bonded and other non-bonded interactions, and the amino acids involved in the interactions are shown in Table 4 for the proteins: IL-6, IL-8, TNF-α and GM-CSF. The ligands docked into target proteins were presented in Figure 10.

The evaluated binding energies ranged from −16.26 to −10.33 kcal/mol. The lowest binding energy was exhibited by 1,6-di-*O*-acetyl-9-deoxyforskolin docked into IL-6, TNF-α and GM-CSF, as well as 1,6-di-*O*-acetylforskolin docked into IL-8. Overall, all ligands docked into IL-8 showed the lowest binding energy. IL-6, IL-8, TNF-α and GM-CSF play significant roles as factors involved in the inflammatory cascade and are specific targets for the molecular docking of various compounds, including those of plant origin.

## 4. Discussion

Since time immemorial, natural products have been the backbone of the traditional healing system around the world, and an integral part of history and culture. Although the use of herbal remedies dates back hundreds or even thousands of years, their use as isolated and characterized compounds did not begin until the 19th century [2,40,41]. Nature has created an almost inexhaustible range of active compounds, and substances of plant origin are of great interest due to their versatility. They are considered an invaluable source of potential therapeutic agents, modern drugs, nutraceuticals, dietary supplements, folk remedies and pharmaceutical intermediates; many are used as precursors to synthetic drugs and modern therapeutic approaches [42,43,44]. It is estimated that about 30% of higher plant species are used in medicine, and that 74% of pharmacologically-active compounds of plant origin were discovered based on their ethnomedical uses [42,45,46].

The aim of this study was to determine the previously unknown biological properties of selected halimane and labdane diterpenes isolated from *Plectranthus ornatus*: (11R*, 13E)-11-acetoxyhali-ma-5,13-dien-15-oic acid [HAL], 1α, 6β-diacetoxy-8α, 13R*-epoxy-14-labden-11-one-Plectrornatine C [PLEC] and the forskolin-like 1:1 mixture of 1,6-di-*O*-acetylforskolin and 1,6-di-*O*-acetyl-9-deoxyforskolin [MRC]. The study also includes a computational analysis to determine their antioxidant and anti-inflammatory potentials.

The diterpenes (C-20) are derived from geranyl-linalyl pyrophosphate or its C-13 allylic isomer, geranyl-geranyl pyrophosphate (GGPP) and their biological activity was found to increase in the presence of a lactone group [47,48]. To date, a considerable number of diterpenoids possessing a labdane skeleton are known. They comprise a decalin system and a C-6 ring or, as in the case of manoyl oxide and its derivatives, a six-membered heterocycle, showing an oxygen atom. In the ring system of labdanes, drawing the substituents below the plane of the ring with a dashed bond indicates α-configuration, while a wedge bond indicates a β-configuration with the substituents above the ring. Labdane diterpenes have five chiral carbon atoms and naturally occur in two enantiomeric series [49,50,51]. In the present study, PLEC and MRC were classified as labdanes, while HAL was classified as a halimane diterpene. Both labdane and halimane diterpenes show a range of antitumor, antibacterial, antiviral, antifungal, antimalarial, anti-inflammatory, anti-ulcerogenic, antihyperlipidemic and hepatogenic activities [49,50,52,53].

The cytotoxic effects of the compounds were assessed on the lung cancer (A549) and leukemia (CCRF-CEM) cell lines, and on the non-cancerous retinal pigment epithelia (ARPE-19) cell line. It was found that the two compounds (HAL and PLEC) showed a cytotoxic effect against the A549 cancer cell line in the tested concentration range, while IC_50_ was not reached for MRC. In turn, only HAL showed a cytotoxic effect against the CCRF-CEM cells. In both cases the best results were displayed for the halimane diterpene. In other studies, various halimane diterpenes isolated from *Nardophyllum bryoides* were found to exhibit moderate cytotoxicity against the human pancreatic adenocarcinoma cell line [54], while a halimane diterpene isolated from *Vellozia kolbekii*, identified as (5R,8R,9S,13R)-halim-1,10-ene-15,16-diol, showed cytotoxic effect against three human cancer cell lines: MDA-MB-435 (melanoma), SF-295 (glioblastoma), and HCT-8 (colon adenocarcinoma) [55]. Lastly, (13R)-13-hydroxy-1(10) 14-ent-halimadien-18-oic acid isolated from *Hymenaea courbaril* exhibited weak cytotoxicity against the A2780 human ovarian cancer cell line [56].

HAL and PLEC, but not MRC, showed significant cytotoxic effects against two other cancer cell lines (FaDu and MCF7) [21]. This variation may result from differences in the spatial distribution of their structures, as well as the different sensitivity of the aforementioned cancer cell lines. Additionally, none of the tested compounds showed a strong cytotoxic effect on normal cells in the tested concentration range, which makes them a good prognostic for further studies, to confirm the promising results herein reported about the activity of these three compounds on CCRF-CEM and A549 cancer cell lines.

The study then examined certain parameters related to apoptosis, such as changes in mitochondrial potential, DNA damage, level of mitochondrial copies and ROS production. MMP is a key indicator of mitochondrial activity, as it reflects the process of electron transport and oxidative phosphorylation, the driving force behind ATP production [57,58]. Loss of mitochondrial membrane potential is a signal of bioenergetic stress and can result in the release of apoptotic factors leading to cell death [59,60]. Changes in mtDNA copy number can alter mitochondrial gene expression and cause abnormal mitochondrial functions such as energy production, signalling, apoptosis, and cell growth. Therefore, abnormal mtDNA content can potentially lead to changes in oxidative phosphorylation and increase the production of ROS in aerobic metabolism [61]. In turn, the blockage of DNA replication by chemical genotoxins can lead to apoptosis, via the breakdown of replication forks and the formation of DNA double-strand breaks (DSBs) [62]. Our studies found that HAL, PLEC and MRC all induced a moderate increase in ROS levels for the A549 line, while only HAL and PLEC exhibited activity for the CCRF-CEM line. 

Additionally, quantum chemical analysis was used to reveal the free radical scavenging activity of the active molecules, a parameter related to the orbital energy boundary charge density distribution (FMO), which explains the electronic nature of the molecules. The HOMO and LUMO play an important role in predicting the charge transfer within the molecule, as well as the chemical reactivity, bioactivity, and stability of the compound. Higher HOMO energy is associated with a stronger electron donor molecule, while LUMO energy reflects the ability to accept the electron. In molecules with antioxidant properties, the distribution of HOMO electron density may qualitatively indicate the active site of free radical scavenging, because the reaction of H-abstraction is associated with the transfer of electrons [63]. 

Our analysis showed that HAL exhibits moderate antioxidant reactivity, with the lowest HOMO-LUMO Egaps value among the analyzed compounds, as confirmed by our ROS studies. Moreover, changes in the apoptotic parameters (MMP, DNA damage or mitochondrial copy number) were only noted for CCRF-CEM, but with a different configuration of the action of the compounds: a decrease in MMP was observed for all tested compounds, as well as an increase in DNA damage for HAL and PLEC, and a change mitochondrial copy number for HAL and MRC. 

Our studies suggest that the CCRF-CEM cancer cell line is more sensitive to the action of the tested compounds; as such, the potential mechanisms of activation of signaling pathways in these cells merit further attention. Previous studies have shown that HAL and PLEC can induce apoptosis in MCF7 and FaDu cells via the mitochondrial pathway [21]. However, the present study demonstrates the apoptotic effect of HAL, PLEC and MRC compounds isolated from *P. ornatus* against CCRF-CEM and A549 cancer cell lines. Previous studies have found another labdane diterpene, andrographolide, to inhibit human hepatoma-derived Hep3B cell growth through the activation of c-Jun N-terminal kinase [64], and the halimane diterpene witextrifloxide G to inhibit the activity of DNA topoisomerase 1 [65]. Due to the limited number of studies showing the induction of apoptosis by compounds belonging to the group of halimane and labdane diterpenes, further studies are necessary to understand their mechanisms of action and other biological properties.

The final step in our study was to check the anti-inflammatory properties of the compounds in a non-cancerous cell line following LPS treatment. Inflammation is a localized protective response by tissue cells to allergic or chemical irritation, trauma or infection. It is associated with pain, warmth, redness, swelling, and loss of function resulting from vasodilation, leading to increased blood supply, and an increase in the intracellular spaces, with consequent movement of leukocytes, proteins and fluids to the inflamed areas [66,67]. Several cytokines, especially interleukin-1 (IL-1) and tumor necrosis factor-a (TNF-a), also play an important role in initiating the inflammatory process. Both are considered to be major mediators of the biological response to bacterial lipopolysaccharide (LPS, also called endotoxin). They are secreted by various cells including monocytes, macrophages and adipocytes [68,69]. Together with various cytokines and growth factors (including IL-8 and the granulocyte-macrophage colony-stimulating factor GM-CSF), they induce gene expression and protein synthesis, thus mediating the onset and development of inflammation [68,70,71]. 

Our findings demonstrated that all the tested halimane and labdane diterpenes HAL, PLEC and MRC possess an anti-inflammatory effect in LPS-induced ARPE-19 cells by reducing the level of expression of *IL-6*, *IL-8*, *TNF-α* and *GM-CSF* genes. Our previous study showed that labdane diterpene PLEC and some halimane derivatives derived from the halimane diterpene A, namely halimane diterpenes B-D, are involved in the cyclooxygenase-2 (COX-2) inhibition [72]. It has been proposed that the labdane diterpene forskolin inhibits the increase in monocyte chemotactic protein-1 (MCP-1) mRNA levels and the decrease in G-protein coupled receptor 120 (GPR120) mRNA levels in adipocytes upon LPS induction, making a potential therapeutic agent for the treatment of inflammation in obesity [73]. 

However, in vivo studies demonstrated that oral administration of forskolin (10 mg/kg) exerts a strong anti-inflammatory effect by reducing paw swelling (87.79%), and that it was comparable to the standard drug indomethacin (10 mg/kg, 93.89%) [74]. In turn, Li et al. revealed that labdane diterpene (10R*,13R*,15R*)-15,16-Epoxy-6,13-dihydroxy-15-methoxy-labda-5,8-dien-7-one inhibited nitric oxide (NO) production by lipopolysaccharide (LPS) in RAW 264.7 cells with an IC_50_ value of 40.1 μM [75]. These findings are consistent with ours and confirm the anti-inflammatory properties of the studied halimane and diterpene labdane compounds. 

Our molecular docking studies evidenced that 1,6-di-*O*-acetyl-9-deoxyforskolin docked to IL-6, TNF-α and GM-CSF and 1,6-di-*O*-acetylforskolin docked at IL-8 have the lowest binding energy of the tested combinations, showing that the ligand is properly absorbed and binds with the target protein; this may confirm that the compounds have an anti-inflammatory effect, which is in line with our gene expression findings. In silico studies conducted by Malik et al. showed that three phytocompounds including isoorientin, lupeol, and andrographolide could have an inhibitory effects on IL-6. Isoorientin, a flavone C-glycoside, showed better binding affinity on IL-6, if compared to all other phytocompounds with a binding energy of −7.7 kcal/mol. Isoorientin with IL-6 interactive residues in molecular interactions were as follow: SER: 91, THR: 92, LEU: 95, PHE: 98, LEU: 140, PHE: 143, LYS: 144, LEU: 147 [76]. In addition, Prathap et al. revealed that stachydrine, a pyrrolidine betaine, binds IL-6 and inhibits it, with a binding energy of −5.9 kcal/mol and hydrogen bonding with GLU: 42 [77]. Docking another phytocompound, the flavonoid quercetin, with IL-8 a binding energy of −7.9 kcal/mol was reported [78]. The docking energy score of stachydrine with TNF-α is −6.8 and hydrogen bonding with ARG: 332, ARG: 372 and VAL: 373 was observed [77]. The andrographolide, a labdane diterpene, binding energy with GM-CSF was −6.2 kcal/mol [79]. When the binding energy is lower than −5.0 kcal/mol, a better binding profile is shown [80]. Thus, according to our study, even the strongest interactions of 1,6-di-*O*-acetyl-9-deoxyforskolin with the targeted proteins including IL-6, TNF-α and GM-CSF, as well as 1,6-di-*O*-acetylforskolin, with IL-8 were revealed.

However, due to the very limited number of studies on the antioxidant and anti-inflammatory properties of the HAL, PLEC and MRC, further work is needed. This work is preliminary, so it is extremely important to explore the activity of these compounds in the future. The research carried out so far has made it possible to initially select potential avenues for supplementary analysis. The aim of subsequent studies should focus on the mechanism of action in order to identify new desirable biological properties of the compounds under investigation. Future research will also aim to evaluate different cancer cell lines and select the most sensitive to the compounds being analyzed. In addition, after the in vitro stage, in vivo studies will be carried out for the selected diterpenes so to assess their impact on living organisms. 

## 5. Conclusions

In conclusion, the tested halimane and labdane diterpenes isolated from *Plectranthus ornatus*. viz. HAL and PLEC showed a cytotoxic effect on leukemia (CCRF-CEM) and lung (A549) cancer cells. In addition, HAL, PLEC and MRC were found to increase ROS levels for the A549 line, while HAL and PLEC induced ROS for the CCRF-CEM cancer cell lines. The in silico analysis based on HOMO/LUMO orbitals indicated that the HAL compound had moderate antioxidant properties. Moreover, all the tested compounds altered the mitochondrial membrane potential (MMP) and mitochondrial copy number, and caused DNA damage, but only in the leukemia CCRF-CEM cancer cell line. All the compounds (HAL, PLEC and MRC) also demonstrated anti-inflammatory effects by decreasing the observed changes in gene expression (IL-6, IL-8, TNF-α and GM-CSF) in ARPE-19 cells upon LPS-induction. An integrated approach of virtual screening and molecular docking provided structural insights into possible binding modes of bioactive compounds of *P. ornatus*. The lowest binding energies were exhibited by 1,6-di-*O*-acetyl-9-deoxyforskolin docked into IL-6, TNF-α and GM-CSF, as well as 1,6-di-*O*-acetylforskolin docked into IL-8. In addition, ADMET studies showed very interesting drug-likeness properties for the analyzed compounds. Overall, these results suggest a fairly high biological potential for the tested compounds, but further studies are necessary to evaluate their pharmacological and toxicity profiles in in vivo models.

## Figures and Tables

**Table 1:** Physicochemical properties, lipophilicity, solubility, bioavailability, and drug-likeness predictions for the plant-derived diterpenes and the positive control, gemcitabine.

Compound Name	HAL	PLEC	1,6-di-*O*-acetylforskolin	1,6-do-*O*-acetyl-9-deoxyforskolin	Gemcitabine
Formula	C_25_H_36_O_5_	C_24_H_36_O_6_	C_26_H_38_O_8_	C_26_H_38_O_9_	C_9_H_11_F_2_N_3_O_4_
MW ^1^ (g/mol)	416.55	420.54	478.58	494.57	236.20
RB ^2^	6	5	7	7	2
HBA ^3^	5	6	8	9	7
HBD ^4^	1	0	0	1	3
Fraction C sp ^3,5^	0.72	0.79	0.77	0.77	0.56
TPSA ^6^ (Å^2^)	80.67	78.90	105.20	125.43	110.60
Log Po/w ^7^	4.22	3.60	3.31	2.68	−1.46
LogS ^8^	−5.32	−4.21	−4.18	−3.80	−0.67
Lipinski ^9^	Yes (0)	Yes (0)	Yes (0)	Yes (0)	Yes (0)
Bioavailability Score ^10^	0.56	0.55	0.55	0.55	0.55

^1^ Molecular weight; ^2^ number of rotatable bonds; ^3^ number of hydrogen bond acceptors; ^4^ number of hydrogen bond donors; ^5^ the ratio of sp^3^ hybridized carbons over the total carbon count of the molecule; ^6^ topological polar surface area; ^7^ the octanol-water partition coefficient; ^8^ LogS, as a measure of solubility. ^9^ Lipinski’s rule of 5 sets 5 criteria; ^10^ probability of a compound to have a bioavailability of more than 10% in rats. All the aforementioned properties were evaluated by SwissADME.

**Table 2:** Pharmacokinetic and toxicity predictions for plant-derived diterpenes and positive control—gemcitabine.

Compound Name	HAL	PLEC	1,6-di-*O*-acetylforskolin	1,6-do-*O*-acetyl-9-deoxyforskolin	Gemcitabine
GI absorption ^1^	High	High	High	High	High
BBB Permeant ^2^	No	No	No	No	No
P-gp Substrate ^3^	Yes	No	No	Yes	No
CYP1A2 inhibitor	No	No	No	No	No
CYP2C19 inhibitor	No	No	No	No	No
CYP2C9 inhibitor	Yes	No	No	No	No
CYP2D6 inhibitor	No	No	No	No	No
CYP3A4 inhibitor	Yes	No	No	Yes	No
Log Kp ^4^ (cm/s)	−5.13	−6.51	−7.15	−7.79	−8.94
LD50 ^5^ (mg/kg)	3300	100	2550	2550	1000
Hepatotoxicity					
Carcinogenicity					
Mutagenicity					
A ^6^					
p ^7^	0.88	0.82	0.75	0.68	0.81
Cytotoxicity					
A ^6^					
p ^7^	0.80	0.78	0.69	0.72	0.94

^1^ Gastrointestinal absorption; ^2^ blood–brain barrier; ^3^ P-glycoprotein; ^4^ measure of skin permeation. The more negative the value of logKp, the lower the skin permeability indicated. All of the aforementioned pharmacokinetic properties were predicted by SwissADME; ^5^ lethal dose, as calculated by ProToxII; ^6^ prediction for the inactivity (green) or activity (red) of the compound in the respective toxicity category, as given by ProToxII; ^7^ probability of this prediction, given by ProToxII.

**Table 3:** Energy of HOMO/LUMO orbitals, Egaps and other reactivity descriptors.

Molecular Descriptor	HAL	1,6-di-*O*-acetylforskolin	1,6-di-*O*-acetyl-9-deoxyforskolin	PLEC	Caffeic Acid (Positive Standard)	Phenol (Negative Standard)
**HOMO energy (eV)**	−6.399	−6.959	−6.782	−6.892	−6.186	−6.238
**LUMO energy (eV)**	−1.605	−1.109	−1.113	−1.013	−2.059	−0.377
**Egaps (eV)**	4.794	5.850	5.669	5.879	4.127	5.861
**IP (eV)**	6.399	6.959	6.782	6.892	6.186	6.238
**EA (eV)**	1.605	1.109	1.113	1.103	2.059	0.377

**Table 4:** Binding energy, hydrogen bonding and other nonbonding interactions in ligands–proteins docking.

Proteins	Plant-Derived Compounds	Binding Free Energy (kcal/mol)	Hydrogen Bonding	Other Interactions
IL-6	HAL	−10.33	GLU A:42 ARG A:104 GLU A:106 ASP A:160	LYS A:46 PHE A:105 THR A:163
	1,6-di-*O*-acetylforskolin	−10.87	-	TYR A:31 ILE A:32 ASP A:34 GLN A:111 VAL A:115
	1,6-di-*O*-acetyl-9-deoxyforskolin	−12.16	-	LYS A:66 GLU A:172 SER A:169
	PLEC	−11.06	-	PRO A:65 SER A:169 GLU A:172
IL-8	HAL	−14.48	ILE A:8 LYS A:9 LEU A:47 CYS A:48	TYR A:11 GLU A:46
	1,6-di-*O*-acetylforskolin	−16.26	ILE A:8 LYS A:9	TYR A:11 GLU A:46 LEU A:47
	1,6-di-*O*-acetyl-9-deoxyforskolin	−15.85	ILE A:8 LYS A:9 ARG A:45	GLU A:46
	PLEC	−14.82	ILE A:8 LYS A:9 GLU A:46	TYR A:11 LEU A:47
TNF-α	HAL	−11.96	-	GLU A:116 PRO C:100 GLU C:116
	1,6-di-*O*-acetylforskolin	−14.12	-	GLU A:116 LYS C:98
	-di-*O*-acetyl-9-deoxyforskolin)	−15.18	GLN A:102 SER B:99 GLN C:102	CYS A:101 GLU A:116 GLU B:116 LYS C:98 TYR C:115 GLU C:116
	PLEC	−13.49	CYS A:69 GLN B:102 GLN C:102	GLU C:116
GM-CSF	HAL	−11.36	TRP B13 GLU B:14 SER B:82 HIS B:83 GLN B:86	PRO B:8 PRO B:6 PRO B:12 HIS B:87
	1,6-di-*O*-acetylforskolin	−13.20	GLN A:86	PRO A:8 PRO A:12 GLU A:14 HIS A:87
	1,6-di-*O*-acetyl-9-deoxyforskolin)	−13.84	TRP A:13 GLN A:86	SER A:8 GLU A:14 SER A:82 HIS A:87
	PLEC	−12.07	GLN A:86	PRO A:8 PRO A:12 GLU A:14

## Data Availability

Not applicable.

## Supplementary Materials

The following supporting information can be downloaded at: https://www.mdpi.com/article/10.3390/cells11203243/s1, Figure S1: Representative images of comets.

## References

[1] Sofowora A., Ogunbodede E., Onayade A. (2013). The Role and Place of Medicinal Plants in the Strategies for Disease Prevention. Afr. J. Tradit. Complement. Altern. Med..

[2] Yuan H., Ma Q., Ye L., Piao G. (2016). The Traditional Medicine and Modern Medicine from Natural Products. Molecules.

[3] Krause J., Tobin G. (2013). Discovery, Development, and Regulation of Natural Products. Using Old Solutions to New Problems—Natural Drug Discovery in the 21st Century.

[4] Barnum C.R., Endelman B.J., Shih P.M. (2021). Utilizing Plant Synthetic Biology to Improve Human Health and Wellness. Front. Plant Sci..

[5] Kowalczyk T., Merecz-Sadowska A., Rijo P., Mori M., Hatziantoniou S., Górski K., Szemraj J., Piekarski J., Śliwiński T., Bijak M. (2022). Hidden in Plants—A Review of the Anticancer Potential of the Solanaceae Family in In Vitro and In Vivo Studies. Cancers.

[6] Merecz-Sadowska A., Sitarek P., Śliwiński T., Zajdel K., Malinowska K., Zielińska-Bliźniewska H., Kucharska E., Zajdel R. (2022). In Vitro and In Silico Studies on *Leonotis Nepetifolia* (L.) R. Br. Root Extract against Cancer Cells. Curr. Pharm. Biotechnol..

[7] Kowalczyk T., Merecz-Sadowska A., Rijo P., Isca V.M.S., Picot L., Wielanek M., Śliwiński T., Sitarek P. (2021). Preliminary Phytochemical Analysis and Evaluation of the Biological Activity of *Leonotis Nepetifolia* (L.) R. Br Transformed Roots Extracts Obtained through *Rhizobium Rhizogenes*-Mediated Transformation. Cells.

[8] Kowalczyk T., Sitarek P., Merecz-Sadowska A., Szyposzyńska M., Spławska A., Gorniak L., Bijak M., Śliwiński T. (2021). Methyl Jasmonate Effect on Betulinic Acid Content and Biological Properties of Extract from *Senna Obtusifolia* Transgenic Hairy Roots. Molecules.

[9] Śliwiński T., Sitarek P., Skała E., Isca V.M.S., Synowiec E., Kowalczyk T., Bijak M., Rijo P. (2020). Diterpenoids from *Plectranthus* Spp. as Potential Chemotherapeutic Agents via Apoptosis. Pharmaceuticals.

[10] Sitarek P., Toma M., Ntungwe E., Kowalczyk T., Skała E., Wieczfinska J., Śliwiński T., Rijo P. (2020). Insight the Biological Activities of Selected Abietane Diterpenes Isolated from *Plectranthus* spp.. Biomolecules.

[11] Rice L.J., Brits G.J., Potgieter C.J., van Staden J. (2011). *Plectranthus*: A Plant for the Future?. South Afr. J. Bot..

[12] Lukhoba C.W., Simmonds M.S.J., Paton A.J. (2006). *Plectranthus*: A Review of Ethnobotanical Uses. J. Ethnopharmacol..

[13] Abdel-Mogib M., Albar H.A., Batterjee S.M. (2002). Chemistry of the Genus Plectranthus. Molecules.

[14] Waldia S., Joshi B.C., Pathak U., Joshi M.C. (2011). The Genus Plectranthus in India and Its Chemistry. Chem. Biodivers..

[15] Nascimento F.R., Albuquerque K.R.S., Oliveira M.R., Pizziolo V.R., Brasileiro B.G., Diaz G., Diaz M.A.N. (2017). Antibiotic Activity of *Plectranthus Ornatus* Codd., a Traditional Medicinal Plant. An. Acad. Bras. Ciências.

[16] Ascensão L., Mota L., Castro M.D.M. (1999). Glandular Trichomes on the Leaves and Flowers of *Plectranthus Ornatus*: Morphology, Distribution and Histochemistry. Ann. Bot..

[17] Passinho-Soares H.C., Meira P.R., David J.P., Mesquita P.R.R., do Vale A.E., de Rodrigues F.M., de Pereira P.A.P., de Santana J.R.F., de Oliveira F.S., de Andrade J.B. (2013). Volatile Organic Compounds Obtained by in Vitro Callus Cultivation of *Plectranthus Ornatus* Codd. (Lamiaceae). Molecules.

[18] Rijo P., Gaspar-Marques C., Simões M.F., Duarte A., Apreda-Rojas M.d.C., Cano F.H., Rodríguez B. (2002). Neoclerodane and Labdane Diterpenoids from *Plectranthus Ornatus*. J. Nat. Prod..

[19] Rijo P., Gaspar-Marques C., Simões M.F., Jimeno M.L., Rodríguez B. (2007). Further Diterpenoids from *Plectranthus Ornatus* and *P. Grandidentatus*. Biochem. Syst. Ecol..

[20] Rijo P., Simões M.F., Rodríguez B. (2005). Structural and Spectral Assignment of Three Forskolin-like Diterpenoids Isolated from *Plectranthus Ornatus*. Magn. Reson. Chem..

[21] Sitarek P., Synowiec E., Kowalczyk T., Bangay G., Śliwiński T., Picot L., Princiotto S., Rijo P. (2022). Anticancer Properties of *Plectranthus ornatus*-Derived Phytochemicals Inducing Apoptosis via Mitochondrial Pathway. Int. J. Mol. Sci..

[22] Reiniers M.J., de Haan L.R., Reeskamp L.F., Broekgaarden M., van Golen R.F., Heger M. (2021). Analysis and Optimization of Conditions for the Use of 2′,7′-Dichlorofluorescein Diacetate in Cultured Hepatocytes. Antioxidants.

[23] Sivandzade F., Bhalerao A., Cucullo L. (2019). Analysis of the Mitochondrial Membrane Potential Using the Cationic JC-1 Dye as a Sensitive Fluorescent Probe. Bio-Protocol.

[24] Bijak M., Synowiec E., Sitarek P., Sliwiński T., Saluk-Bijak J. (2017). Evaluation of the Cytotoxicity and Genotoxicity of Flavonolignans in Different Cellular Models. Nutrients.

[25] Ceremuga M., Stela M., Janik E., Gorniak L., Synowiec E., Sliwinski T., Sitarek P., Saluk-Bijak J., Bijak M. (2020). Melittin—A Natural Peptide from Bee Venom Which Induces Apoptosis in Human Leukaemia Cells. Biomolecules.

[26] Sitarek P., Synowiec E., Kowalczyk T., Śliwiński T., Skała E. (2018). An In Vitro Estimation of the Cytotoxicity and Genotoxicity of Root Extract from *Leonurus Sibiricus* L. Overexpressing AtPAP1 against Different Cancer Cell Lines. Molecules.

[27] Singh N.P., McCoy M.T., Tice R.R., Schneider E.L. (1988). A Simple Technique for Quantitation of Low Levels of DNA Damage in Individual Cells. Exp. Cell Res..

[28] Klaude M., Eriksson S., Nygren J., Ahnström G. (1996). The Comet Assay: Mechanisms and Technical Considerations. Mutat. Res..

[29] Smith C.C., O’Donovan M.R., Martin E.A. (2006). HOGG1 Recognizes Oxidative Damage Using the Comet Assay with Greater Specificity than FPG or ENDOIII. Mutagenesis.

[30] Schmittgen T.D., Livak K.J. (2008). Analyzing Real-Time PCR Data by the Comparative CT Method. Nat. Protoc..

[31] Snyder H.D., Kucukkal T.G. (2021). Computational Chemistry Activities with Avogadro and ORCA. J. Chem. Educ..

[32] Hunter A.D. (1997). ACD/ChemSketch 1.0 (Freeware); ACD/ChemSketch 2.0 and Its Tautomers, Dictionary, and 3D Plug-Ins; ACD/HNMR 2.0; ACD/CNMR 2.0. J. Chem. Educ..

[33] O’Boyle N.M., Banck M., James C.A., Morley C., Vandermeersch T., Hutchison G.R. (2011). Open Babel: An Open Chemical Toolbox. J. Cheminform..

[34] Morris G.M., Ruth H., Lindstrom W., Sanner M.F., Belew R.K., Goodsell D.S., Olson A.J. (2009). AutoDock4 and AutoDockTools4: Automated Docking with Selective Receptor Flexibility. J. Comput. Chem..

[35] Morris G.M., Goodsell D.S., Halliday R.S., Huey R., Hart W.E., Belew R.K., Olson A.J. (1998). Automated Docking Using a Lamarckian Genetic Algorithm and an Empirical Binding Free Energy Function. J. Comput. Chem..

[36] Coimbra J.T.S., Feghali R., Ribeiro R.P., Ramos M.J., Fernandes P.A. (2021). The Importance of Intramolecular Hydrogen Bonds on the Translocation of the Small Drug Piracetam through a Lipid Bilayer. RSC Adv..

[37] Daina A., Michielin O., Zoete V. (2017). SwissADME: A Free Web Tool to Evaluate Pharmacokinetics, Drug-likeness and Medicinal Chemistry Friendliness of Small Molecules. Sci. Rep..

[38] Seelig A. (2020). P-Glycoprotein: One Mechanism, Many Tasks and the Consequences for Pharmacotherapy of Cancers. Front. Oncol..

[39] Purnapatre K., Khattar S.K., Saini K.S. (2008). Cytochrome P450s in the development of target-based anticancer drugs. Cancer Lett..

[40] Veeresham C. (2012). Natural Products Derived from Plants as a Source of Drugs. J. Adv. Pharm. Technol. Res..

[41] Lautié E., Russo O., Ducrot P., Boutin J.A. (2020). Unraveling Plant Natural Chemical Diversity for Drug Discovery Purposes. Front. Pharmacol..

[42] Atanasov A.G., Waltenberger B., Pferschy-Wenzig E.M., Linder T., Wawrosch C., Uhrin P., Temml V., Wang L., Schwaiger S., Heiss E.H. (2015). Discovery and Resupply of Pharmacologically Active Plant-Derived Natural Products: A Review. Biotechnol. Adv..

[43] Howes M.J.R., Quave C.L., Collemare J., Tatsis E.C., Twilley D., Lulekal E., Farlow A., Li L., Cazar M.E., Leaman D.J. (2020). Molecules from Nature: Reconciling Biodiversity Conservation and Global Healthcare Imperatives for Sustainable Use of Medicinal Plants and Fungi. Plants People Planet.

[44] Pan S.Y., Zhou S.F., Gao S.H., Yu Z.L., Zhang S.F., Tang M.K., Sun J.N., Ma D.L., Han Y.F., Fong W.F. (2013). New Perspectives on How to Discover Drugs from Herbal Medicines: CAM’s Outstanding Contribution to Modern Therapeutics. Evid. Based Complement. Altern. Med..

[45] Bhat S.G. (2021). Medicinal Plants and Its Pharmacological Values. Natural Medicinal Plants.

[46] Stéphane F.F.Y., Jules B.K.J., Batiha G.E.-S., Ali I., Bruno L.N. (2021). Extraction of Bioactive Compounds from Medicinal Plants and Herbs. Natural Medicinal Plants.

[47] Demetzos C., Dimas K.S. (2001). Labdane-Type Diterpenes: Chemistry and Biological Activity. Stud. Nat. Prod. Chem..

[48] Mani V., Park S., Kim J.A., Lee S.I., Lee K. (2021). Metabolic Perturbation and Synthetic Biology Strategies for Plant Terpenoid Production—An Updated Overview. Plants.

[49] Silva L., Gomes A.C. (2011). Diterpene Lactones with Labdane, Halimane and Clerodane Frameworks. Nat. Prod. Commun..

[50] Roncero A.M., Tobal I.E., Moro R.F., Díez D., Marcos I.S. (2018). Halimane Diterpenoids: Sources, Structures, Nomenclature and Biological Activities. Nat. Prod. Rep..

[51] Majhi S. (2020). Diterpenoids: Natural Distribution, Semisynthesis at Room Temperature and Pharmacological Aspects—A Decade Update. ChemistrySelect.

[52] Saha P., Rahman F.I., Hussain F., Rahman S.M.A., Rahman M.M. (2022). Antimicrobial Diterpenes: Recent Development From Natural Sources. Front. Pharmacol..

[53] Seaman F., Bohlmann F., Zdero C., Mabry T.J. (1990). Biological Activity of Diterpenes. Diterpenes Flower. Plants.

[54] Sánchez M., Mazzuca M., Veloso M.J., Fernández L.R., Siless G., Puricelli L., Palermo J.A. (2010). Cytotoxic Terpenoids from Nardophyllum Bryoides. Phytochemistry.

[55] Silva C.G., Santos H.M., Barbosa J.P., Costa G.L., Rodrigues F.A.R., Oliveira D.F., Costa-Lotufo L.V., Alves R.J.V., Eleutherio E.C.A., Rezende C.M. (2015). Structure Elucidation, Antimicrobial and Cytotoxic Activities of a Halimane Isolated from Vellozia Kolbekii Alves (Velloziaceae). Chem. Biodivers..

[56] Abdel-Kader M., Berger J.M., Slebodnick C., Hoch J., Malone S., Wisse J.H., Werkhoven M.C.M., Mamber S., Kingston D.G.I. (2001). Isolation and Absolute Configuration of Ent-Halimane Diterpenoids from Hymenaea Courbaril from the Suriname Rain Forest1. J. Nat. Prod..

[57] Zorova L.D., Popkov V.A., Plotnikov E.Y., Silachev D.N., Pevzner I.B., Jankauskas S.S., Babenko V.A., Zorov S.D., Balakireva A.V., Juhaszova M. (2018). Mitochondrial Membrane Potential. Anal. Biochem..

[58] Kühlbrandt W. (2015). Structure and Function of Mitochondrial Membrane Protein Complexes. BMC Biol..

[59] Wang C., Youle R.J. (2009). The Role of Mitochondria in Apoptosis*. Annu. Rev. Genet..

[60] Burke P.J. (2017). Mitochondria, Bioenergetics and Apoptosis in Cancer. Trends Cancer.

[61] Hu L., Yao X., Shen Y. (2016). Altered Mitochondrial DNA Copy Number Contributes to Human Cancer Risk: Evidence from an Updated Meta-Analysis. Sci. Rep..

[62] Roos W.P., Kaina B. (2006). DNA Damage-Induced Cell Death by Apoptosis. Trends Mol. Med..

[63] Spiegel M. (2022). Current Trends in Computational Quantum Chemistry Studies on Antioxidant Radical Scavenging Activity. J. Chem. Inf. Model..

[64] Ji L., Liu T., Liu J., Chen Y., Wang Z. (2007). Andrographolide Inhibits Human Hepatoma-Derived Hep3B Cell Growth through the Activation of c-Jun N-Terminal Kinase. Planta Med..

[65] Luo P., Yu Q., Liu S.N., Xia W.J., Fang Y.Y., An L.K., Gu Q., Xu J. (2017). Diterpenoids with Diverse Scaffolds from Vitex Trifolia as Potential Topoisomerase I Inhibitor. Fitoterapia.

[66] Chen L., Deng H., Cui H., Fang J., Zuo Z., Deng J., Li Y., Wang X., Zhao L. (2017). Inflammatory Responses and Inflammation-Associated Diseases in Organs. Oncotarget.

[67] Das U.N. (2011). Inflammation. Molecular Basis of Health and Disease.

[68] Kany S., Vollrath J.T., Relja B. (2019). Cytokines in Inflammatory Disease. Int. J. Mol. Sci..

[69] Parameswaran N., Patial S. (2010). Tumor Necrosis Factor-α Signaling in Macrophages. Crit. Rev. Eukaryot. Gene Expr..

[70] McQualter J.L., Darwiche R., Ewing C., Onuki M., Kay T.W., Hamilton J.A., Reid H.H., Bernard C.C.A. (2001). Granulocyte Macrophage Colony-Stimulating Factor: A New Putative Therapeutic Target in Multiple Sclerosis. J. Exp. Med..

[71] Xu Y., Hunt N.H., Bao S. (2008). The Role of Granulocyte Macrophage-Colony-Stimulating Factor in Acute Intestinal Inflammation. Cell Res..

[72] Repositório Da Universidade de Lisboa: Phytochemical Study and Biological Activities of Diterpenes and Derivatives from Plectranthus Species. https://repositorio.ul.pt/handle/10451/2833.

[73] Chiadak J.D., Arsenijevic T., Verstrepen K., Gregoire F., Bolaky N., Delforge V., Flamand V., Perret J., Delporte C. (2016). Forskolin Inhibits Lipopolysaccharide-Induced Modulation of MCP-1 and GPR120 in 3T3-L1 Adipocytes through an Inhibition of NFκ B. Mediators Inflamm..

[74] Karthika K., Jamuna S., Abinaya G., Venkatachalapathi A., Thenmozhi K., Paulsamy S. (2016). Evaluation of Anti-Inflammatory and Antioxidant Properties of Crude Extract and Forskolin from Solena Amplexicaulis Leaf. Indian J. Pharm. Sci..

[75] Li H.Y., Li Y., Wei W.J., Ma K.L., Chen J.J., Gao K. (2020). Halimane and Labdane Diterpenoids from *Leonurus Japonicus* and Their Anti-Inflammatory Activity. Phytochemistry.

[76] Malik A., Naz A., Ahmad S., Hafeez M., Awan F.M., Jafar T.H., Zahid A., Ikram A., Rauff B., Hassan M. (2021). Inhibitory Potential of Phytochemicals on Interleukin-6-Mediated T-Cell Reduction in COVID-19 Patients: A Computational Approach. Bioinform. Biol. Insights.

[77] Prathap L., Jayaraman S., Roy A., Santhakumar P., Jeevitha M. (2021). Molecular docking analysis of stachydrine and sakuranetin with IL-6 and TNF-α in the context of inflammation. Bioinformation.

[78] Shen L., Jiang Y., Lu J., Wang G., Zhang X., He S., Wang C., Li Z. (2021). Molecular Mechanism of Jinchan Oral Liquid in the Treatment of Children with Respiratory Syncytial Virus Pneumonia Based on Network Pharmacology and Molecular Docking Technology. Biomed Res. Int..

[79] Alzahrani A.A. (2022). New Investigation into the Molecular Mechanism of Andrographolide towards Reducing Cytokine Storm. Molecules.

[80] Li C., Du X., Liu Y., Liu Q.Q., Zhi W.B., Wang C.L., Zhou J., Li Y., Zhang H. (2020). A Systems Pharmacology Approach for Identifying the Multiple Mechanisms of Action for the Rougui-Fuzi Herb Pair in the Treatment of Cardiocerebral Vascular Diseases. Evid. Based Complement. Altern. Med..

