# Supplementary figures and images for: An Evaluation of the Novel Biological Properties of Diterpenes Isolated from Plectranthus ornatus Codd. In Vitro and In Silico
Source: Cells. 2022 Oct 15;11(20):3243. doi: 10.3390/cells11203243 (PMC9600095; doi:10.3390/cells11203243)

A549

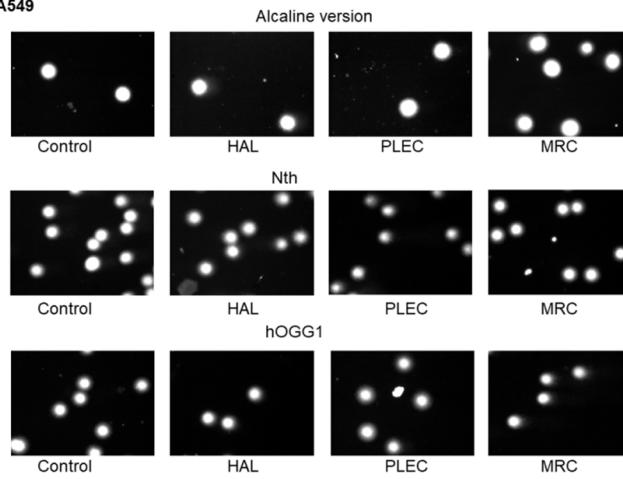

CCRF-CEM

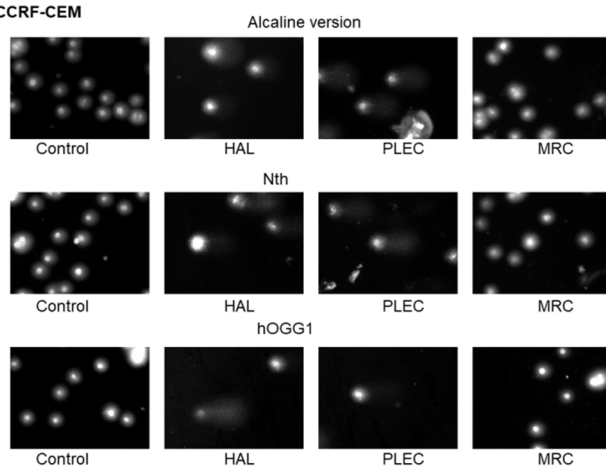

Figure S1: Representative images of comets.

Supplement: Supplementary file 1 [file cells-11-03243-s001.zip › cells-1914070-supplementary.pdf]
